# XPS and HR TEM Elucidation of the Diversity of Titania-Supported Single-Site Ir Catalyst Performance in Spin-Selective Propene Hydrogenation

**DOI:** 10.3390/ijms242115643

**Published:** 2023-10-27

**Authors:** Anna V. Nartova, Ren I. Kvon, Larisa M. Kovtunova, Ivan V. Skovpin, Igor V. Koptyug, Valerii I. Bukhtiyarov

**Affiliations:** 1 Department of Physical-Chemical Methods of Investigation, Boreskov Institute of Catalysis SB RAS, Lavrentiev Ave. 5, 630090 Novosibirsk, Russia; kvon@catalysis.ru (R.I.K.); vib@catalysis.ru (V.I.B.); 2 Laboratory of Magnetic Resonance Microimaging, International Tomography Center SB RAS, Institutskaya St. 3A, 630090 Novosibirsk, Russiakoptyug@tomo.nsc.ru (I.V.K.)

**Keywords:** XPS, NMR, heterogeneous catalysis, hydrogenation, single-site catalysts

## Abstract

Immobilized [Ir(COD)Cl]_2_-Linker/TiO_2_ catalysts with linkers containing Py, P(Ph)_2_ and N(CH_3_)_2_ functional groups were prepared. The catalysts were tested via propene hydrogenation with parahydrogen in a temperature range from 40 °C to 120 °C which was monitored via NMR. The catalytic behavior of [Ir(COD)Cl]_2_-Linker/TiO_2_ is explained on the basis of quantitative and qualitative XPS data analysis performed for the catalysts before and after the reaction at 120 °C. It is shown that the temperature dependence of propene conversion and the enhancement of the NMR signal are explained via a combination of the stabilities of both the linker and immobilized [Ir(COD)Cl]_2_ complex. It is demonstrated that the N(CH_3_)_2_-linker is the most stable at the surface of TiO_2_ under used reaction conditions. As a result, only this sample shows a rise in the enhancement of the NMR signal in the 100–120 °C temperature range.

## 1. Introduction

The intensive investigation of immobilized metal complexes began in the 1960s. Such complexes were reported to activate most simple molecules, including nitrogen, and to carry out catalytic reactions under milder conditions. However, the use of homogeneous catalysts comes with a number of disadvantages. The main one is the need to separate the homogeneous catalyst from the reaction mixture/products. To solve this problem, the heterogenization of homogeneous catalysts by attaching metal complexes on the surface of a support has been proposed [1]. 

So-called single-site catalysts or immobilized catalysts, with the complex of the transition element anchored to the surface of the support via the linker, can bridge the gap between homogeneous and heterogeneous catalysis to combine their individual advantages and overcome their shortcomings, preserving or improving specificity and selectivity as compared to those of the original homogeneous catalysts [2,3,4,5,6]. For this purpose, the nature of a central atom, ligands and linkers as well as a support can be varied. Of course, such usage of immobilized complexes may introduce another issue, e.g., the washing away of the catalyst by the reaction mixture. One of the ways to overcome this is the application of polydentate ligands capable of being coordinated by two, three or even four atoms to the transition metal while a linker group connects the metal to the support [7]. 

Nuclear magnetic resonance (NMR) is perfectly suitable for the investigation of transition metal complexes [2,3,8]. This becomes particularly important in the context of obtaining parahydrogen-induced polarization (PHIP) [9], where the catalyst plays a key role [2,3,8,10,11]. Indeed, an inalienable condition for the occurrence of PHIP is the preservation of the spin correlation of the two atoms of a parahydrogen molecule (p-H_2_). This is realized if both atoms of one p-H_2_ molecule are attached to the same substrate molecule and it often results in a strong enhancement of the NMR signals of reaction products. It should be mentioned that the added protons would have a characteristic shape of line in the ^1^H NMR spectra, which allows them to be unambiguously identified in the product molecule. The ability of transition metal complexes to produce PHIP effects in the homogeneous hydrogenation of unsaturated hydrocarbons with p-H_2_ in solution is well established [3]. Since such complexes are well known as excellent homogeneous catalysts of hydrogenation, their immobilization on a suitable porous solid support looks promising for practical use [12,13,14,15]. At the same time, for such single-site catalysts, the preservation of the nature of the active site of homogeneous precursors is assumed. Therefore, high values of selectivity in the pairwise addition of molecular hydrogen to a substrate molecule are to be expected [2]. It was shown earlier that iridium complex [Ir(COD)(μ-Cl)]_2_ immobilized on the surface of silica via covalent bonding with a -PPh_2_ group of the linker demonstrates ca. 10% selectivity toward pairwise H_2_ addition [2,4].

However, the determination of both the structure of immobilized catalysts and the intermediates formed during the catalytic cycle remains an extremely difficult challenge. As a rule, this is conducted based on data obtained for homogeneous analogs [16], assuming either the preservation or a minor change in the original structure of the immobilized complex [17,18,19].

For the synthesis of such immobilized catalysts, solid porous supports modified via the grafting of surface linkers with phosphino, amino or other suitable functional groups can be used [20,21,22]. It was shown that the catalytic behavior of single-site catalysts in hydrogenations with parahydrogen depends on the linkers used for the anchoring of the complexes, both due to the nature of the linker and due to the stability of the system [5]. Presumably, alkene conversion is predominantly driven by the formation of metal nanoparticles of the active component due to the decomposition of the immobilized complex while the intact immobilized complex is responsible for the enhancement of NMR signals [2,4]. Then, additional methods should be used to support NMR data to explain the results of catalytic tests.

X-ray photoelectron spectroscopy (XPS), as the method sensitive to the chemical state of elements at the surface, appears to be very useful for the investigation of the surface of single-site catalysts for the explanation of their catalytic behavior [23,24]. Indeed, the determination of the charge state of an element allows one to determine the evolution of an immobilized complex on the support surface at the stage of catalyst preparation and during catalytic cycles.

While the XPS study of immobilized complexes and their precursors is a challenging task in itself [25,26,27], overcoming the methodological issues and reconciling reaction data with XPS data analysis looks very promising for the understanding of obtained NMR results. The comparison of XPS data with the results of NMR studies of the reaction can be a key to understanding the evolution of the system from the immobilization stage to the activation/deactivation of the catalyst.

In the present work, three different patterns of catalytic behavior of [Ir(COD)Cl]_2_-Linker/TiO_2_ catalysts (where the linkers are 2-(4-pyridylethyl)triethoxysilane, 2-(diphenylphosphino)ethyltriethoxysilane and (3-N,N-dimethylaminopropyl)triethoxysilane, designated thereinafter as Py-, P- and N-, correspondingly) in propene hydrogenation in the temperature range of 40–120 °C, monitored via NMR, are explained based on the analysis of XPS data. High-resolution transmission electron microscopy (TEM) and energy-dispersive X-ray spectroscopy (EDX) are used to support the interpretation of XPS observations. A comparison of the linker’s stability and [Ir(COD)Cl]_2_ immobilized complex stability is carried out on the basis of both qualitative and quantitative XPS analysis.

## 2. Results and Discussion

### 2.1. Catalytic Tests

**Figure 1:**
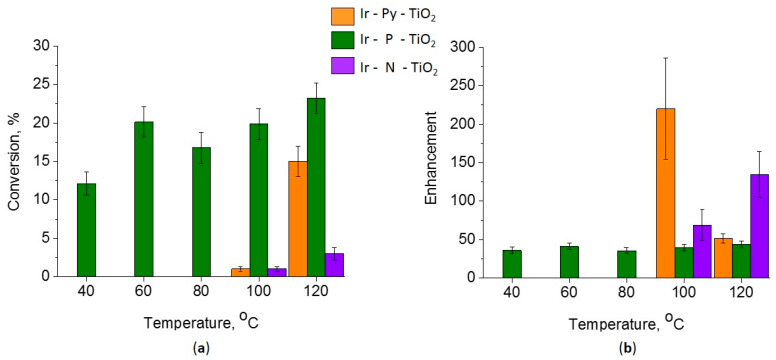
Results of catalytic tests in propene hydrogenetion reaction (**a**) conversion; (**b**) NMR signal enhancement.

For the catalytic reaction tests, propene hydrogenation with p-H_2_ was used (NMR experimental data are shown in Figures S1–S3). In Figure 1, the conversion and enhancement of NMR signal of the methyl group of propane at various reaction temperatures are shown. 

For the Ir-P-TiO_2_ catalyst, the ^1^H NMR signal of propane is seen to start from 40 °C and the catalytic activity of this catalyst is the highest for the investigated set of catalysts (Figure 1). The increase in the total conversion of propene in the range of 40–120 °C is from 12% to 23%, and the temperature dependence of conversion is rather weak. The enhancement, measured for the Ir-P-TiO_2_ sample, varies insignificantly with the reaction temperature and stays in the range of 36–44. The absence of well-pronounced temperature dependence is not typical for such systems and requires further elucidation. At the same time, the enhancement of the propane signal is close to that observed for the hydrogenation of propene on an immobilized iridium complex on SiO_2_, where the enhancements are also in the range of 30–40 [4], and the lower conversion of propene on Ir-P-TiO_2_ is associated with a smaller number of immobilized centers on TiO_2_. 

In contrast, for Ir-Py-TiO_2_ and Ir-N-TiO_2_ catalysts, both activity and enhancement are found only at 100 °C and 120 °C. The significant increase in the conversion up to 15% for the Ir-Py-TiO_2_ sample points to the formation of metal particles above 100 °C, which explains the drop in enhancement from 220 at 100 °C to 51 at 120 °C due to Ir complex decomposition. Note that the signal enhancement for the Ir-Py-TiO_2_ catalyst is comparable to that of the Ir-NH_2_-Linker-SiO_2_ catalyst [4], while the latter is more stable and non-reduced at 120 °C. For the Ir-N-TiO_2_ sample, an analysis of the catalytic tests shows a slight increase in activity along with a pronounced increase in enhancement from 69 at 100 °C to 134 at 120 °C, which implies that the immobilized complex is stable in this temperature range. 

To elucidate the reasons of the unexpected temperature independence of the activity of the Ir-P-TiO_2_ sample, the other differences in catalytic behavior and the impact of stability/decomposition of the immobilized Ir complexes, additional investigations are required. 

### 2.2. XPS Study

Samples after preparation (‘as is’) and after propene hydrogenation at 120 °C were studied via XPS (figures in below and Figures S5–S10).

The ratio of ‘Linker/Ti’ (ratios ‘Linker/Ti’ and ‘Ir/Linker’ were calculated as atomic ratios of N/Ti or P/Ti and Ir/N or Ir/P and for corresponding samples) is considered a measure of the amount of the linker anchored to the surface of TiO_2_. The alteration of the ‘Linker/Ti’ and ‘Ir/Linker’ (total Ir, calculated via the summation of all states of Ir) ratios before and after the reaction unambiguously points to the catalyst’s instability under reaction conditions. The decrease in the Ir/Ti ratio (total Ir) after the reaction compared with that of the ‘as is’ sample can be interpreted either as a removal of the Ir from the surface of the catalyst during the reaction or as the sintering of Ir after immobilized complex decomposition. The sintering of the particles leads to a drop in the Ir 4f line intensity due to a self-screening effect and an increase in the Ti 2p line intensity due to the partial removal of the adsorbed layer from the TiO_2_ surface [24]. 

The ratio of Ir/Cl (total Ir) can be considered an indicator of the undamaged state of immobilized [Ir(COD)Cl]_2_ at the surface of the silica support modified with the linkers [2,26]. Then, the increase in the Ir/Cl ratio after the reaction indicates the decomposition of the complex. In the case of the SiO_2_ support, this assumption works precisely, but for titania, some Cl^−^ ions are able to bind with the support [28,29]. Since Cl 2p binding energy values for this state and for the immobilized [Ir(COD)Cl]_2_ complex are reported to be within the same range of 198.0–198.3 eV, the amount of Cl cannot serve as a strict criterion for complex stability or decomposition over the surface of the TiO_2_ support. 

As expected, the comparison of TiO_2_ modified with corresponding linkers and as-prepared samples of the catalysts shows the stability of the linker during Ir complex immobilization. 

#### 2.2.1. Ir-Py-TiO_2_

**Figure 2:**
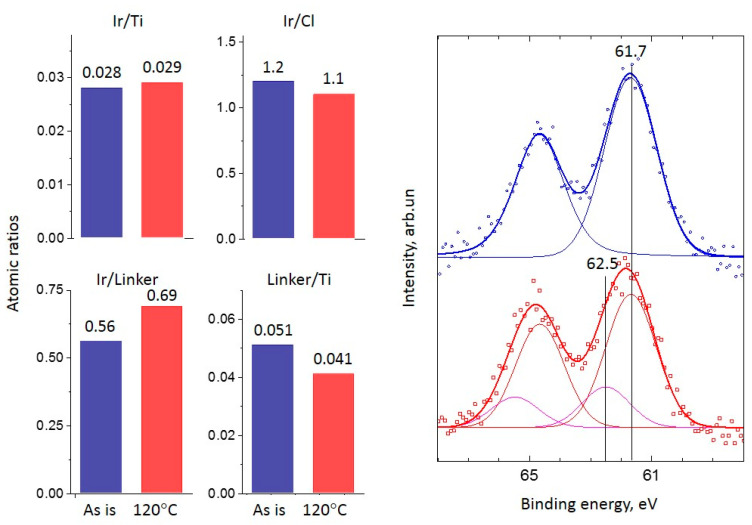
XPS atomic ratios (left panel) (blue bars are for ‘as is’ sample, red bars are for sample after reaction at 120 °C) and spectra (right panel) (points) of the Ir 4f line and spectrum deconvolutions (solid lines, different chemical states are marked by color and with references with binding energy) before (**top blue spectrum**) and after (**bottom red spectrum**) the propene hydrogenation reaction at 120 °C for the Ir-Py-TiO_2_ sample.

For the Ir-Py-TiO_2_ sample, the ‘Linker/Ti’ ratio drops and the ‘Ir/Linker’ ratio rises after the reaction, which can be explained by the partial elimination of the linker from the surface of TiO_2_. At the same time, the total Ir/Ti is constant within the XPS experiment error range. In Figure 2, the Ir 4f line for the Ir-Py-TiO_2_ sample is shown. For the ‘as is’ sample, one state of Ir 4f_7/2_ with a binding energy of 61.7 eV, attributed to Ir(I) from the immobilized [Ir(COD)Cl]_2_ complex [2,4,30], is found. After the reaction, an additional Ir state with a binding energy of 62.5 eV (i.e., Ir(III)) appears [26,27,31]. The formation of Ir(III) is the result of the substantial—about 22%—decomposition of the [Ir(COD)Cl]_2_ complex with the formation of highly dispersed metal Ir species under reductive reaction conditions, accompanied by linker removal from the surface of the support and the subsequent re-oxidation of metal Ir clusters to Ir(III) during the air transfer of the sample into an XPS instrument. Thus, Ir/Ti is stable and ‘Linker/Ti’ and ‘Linker/Ir’ both drop, as was found in the experiment. The considerable drop in the enhancement of the NMR signal (from 220 at 100 °C to 51 at 120 °C) is associated with the partial removal of the linker accompanied by decomposition of the immobilized complex (Figure 1b). At the same time, the formation of the metal Ir leads to an increase in propene conversion up to 15% at 120 °C (Figure 1a).

#### 2.2.2. Ir-P-TiO_2_

**Figure 3:**
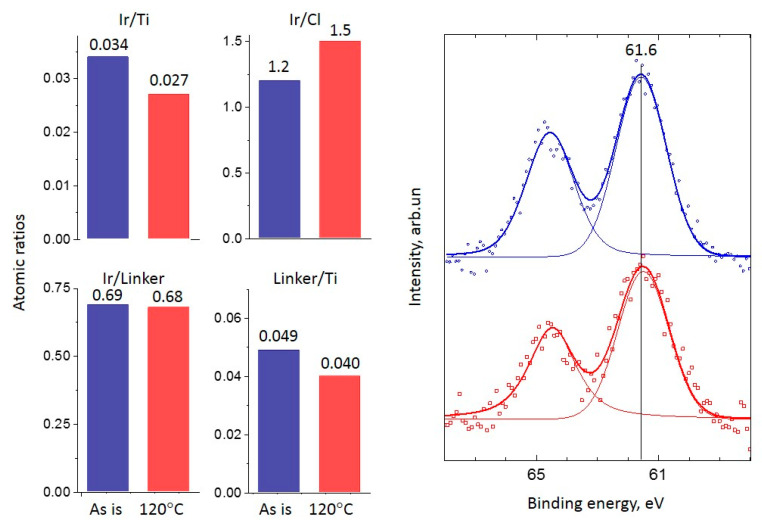
XPS atomic ratios (left panel) (blue bars are for ‘as is’ sample, red bars are for sample after reaction at 120 °C) and spectra (right panel) (points) of the Ir 4f line and spectrum deconvolutions (solid lines, chemical states are marked with reference with binding energy) before (**top blue spectrum**) and after (**bottom red spectrum**) the propene hydrogenation reaction at 120 °C for the Ir-P-TiO_2_ sample.

**Figure 4:**
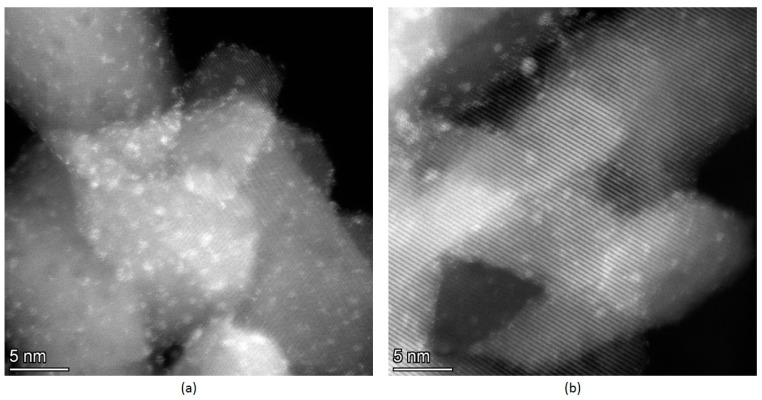
TEM data with Ir clusters. (**a**) ‘as is’ Ir-P-TiO_2_ sample; (**b**) Ir-P-TiO_2_ after reaction at 120 °C.

**Figure 5:**
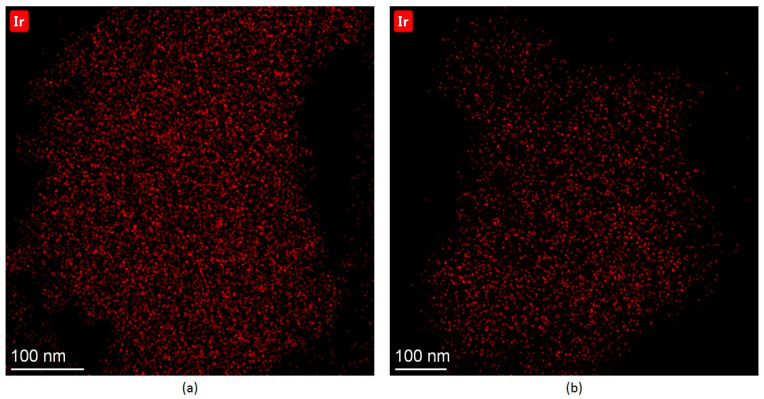
EDX data. (**a**) ‘as is’ Ir-P-TiO_2_ sample; (**b**) Ir-P-TiO_2_ after reaction at 120 °C.

For the Ir-P-TiO_2_ sample after the reaction, a similar decrease in Ir/Ti and Linker/Ti is found while the Ir/Linker ratio does not change (Figure 3). Hence, it can be assumed that the linker leaves the surface together with the Ir complex with its partial decomposition/transformation (Ir/Cl slightly increases). Since the decrease in the Ir/Ti ratio precisely correlates with the eliminated fraction of the linker, regardless of complex decomposition, considerable Ir sintering is not expected. The removal of Cl after the reaction may indicate the rearrangement of the Ir complex at the surface. In Figure 3, the Ir 4f line for the Ir-P-TiO_2_ sample is shown. It is clearly seen that the Ir 4f_7/2_ line is at 61.6–61.5 eV and the shape of the line is the same before and after the reaction. This state is attributed to the immobilized [Ir(COD)Cl]_2_ complex [2,30]. The observed shift can be ascribed to partial complex decomposition or a transformation of complex configuration. The intensity of the Ir 4f line slightly reduces after the reaction. The observations made confirm the assumption about the removal of the Ir complex from the support together with the linker. The apparent stability of the Ir-P-TiO_2_ sample under reaction conditions in the range of 40–120 °C (see Figure 1) can then be explained as a result of the simultaneous increase in the catalytic activity as the temperature is raised and a reduction in the number of active centers as the complex is progressively removed from the surface of TiO_2_. Pronounced propene conversion, shown for the Ir-P-TiO_2_ sample starting from 40 °C (see Figure 1), points to the presence of a specific Ir state which is different from that of other immobilized complexes. This can be Ir in a metal state. Since the binding energy difference for the Ir 4f_7/2_ line for Ir(I) and Ir(0) [23,25,27] is small, and the XPS particle size effect can make it even smaller, the identification of Ir(0) can be impeded by XPS. To support XPS, TEM data before and after the reaction at 120 °C for this sample are shown in Figure 4. Ir clusters or aggregates are clearly seen both before and after the reaction at 120 °C. A comparison of EDX data (see Figure 5) shows a uniform distribution of Ir over the support before and after the reaction. The coverage of the surface with Ir aggregates seen in TEM suggests that the contribution of the state of Ir should be easily seen in XPS. Since the analysis of XPS spectra did not reveal any considerable contributions of Ir(0) or Ir(III), it can be proposed that clusters are made of immobilized complexes. These clusters are apparently responsible for the conversion of propene. It is important that the mean size of such clusters is 0.7 nm and is similar before and after the reaction, i.e., sintering is not detected. Thus, TEM also confirms the assumption made earlier about the elimination of the Ir complex together with the linker rather than sintering.

#### 2.2.3. Ir-N-TiO_2_

**Figure 6:**
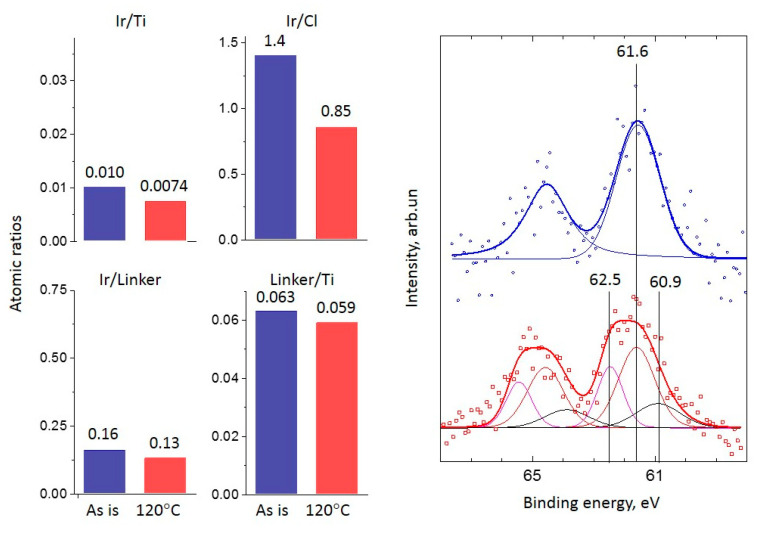
XPS atomic ratios (left panel) (blue bars are for ‘as is’ sample, red bars are for sample after reaction at 120 °C) and spectra (right panel) (points) of the Ir 4f line and spectrum deconvolutions (solid lines, different chemical states are marked by color and with references with binding energy) before (**top blue spectrum**) and after (**bottom red spectrum**) the propene hydrogenation reaction at 120 °C for the Ir-N-TiO_2_ sample.

**Figure 7:**
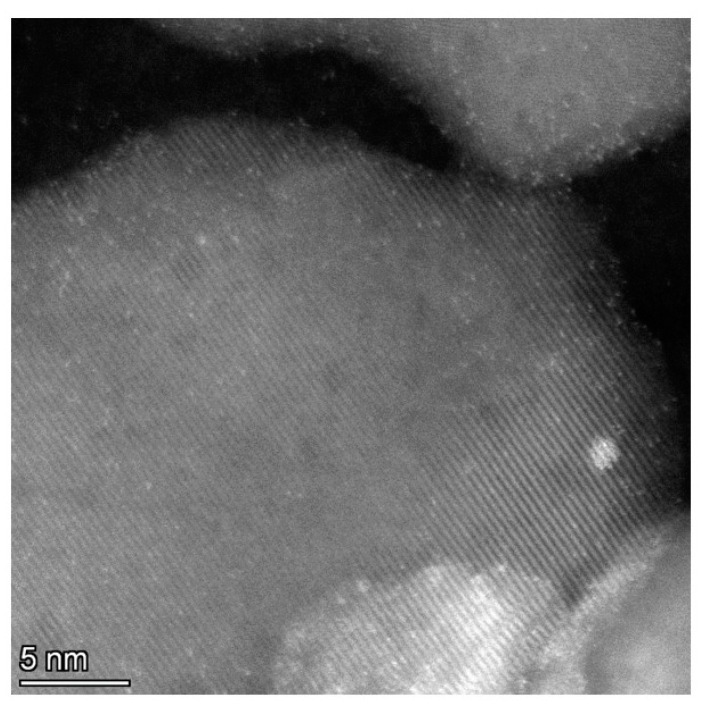
TEM data for the Ir-N-TiO_2_ sample after the reaction at 120 °C.

For the Ir-N-TiO_2_ sample, the ‘Linker/Ti’ ratio is rather stable, while that of ‘Ir/Linker’ slightly decreases because of the drop in Ir content (Figure 6). Compared with Py and P linkers, the N linker is the most stable under propene hydrogenation conditions. Regardless of linker stability at the surface of TiO_2_, the drop in the Ir/Ti ratio can be explained by the partial decomposition of the immobilized [Ir(COD)Cl]_2_ complex. In Figure 6, the Ir 4f line for the Ir-N-TiO_2_ sample is shown. For the ‘as is’ sample, a single state with a Ir 4f_7/2_ binding energy of 61.6 eV is found. Despite the low intensity of the Ir 4f line, after the reaction it is clearly seen that the shape of the line changes (the line width doubles) and its intensity decreases. The deconvolution of the spectrum after the reaction reveals three co-existing states: a state with a binding energy of Ir 4f_7/2_ of 60.9 eV attributed to Ir(0) [23,25,27], of 61.6 eV attributed to Ir(I) (complex) and of 62.5 eV attributed to Ir(III) (oxidized highly dispersed iridium). If the drop in the Ir content was caused by the removal of the complex, only Ir(I) would be seen in the XPS spectrum. The observation of the metal state of Ir indicates the partial decomposition of the complex followed by the migration and aggregation of the obtained individual Ir atoms to yield metal particles that are more stable than individual atoms against oxidation in air during transfer to the XPS spectrometer [32]. The increase in the enhancement of the NMR signal from 69 to 134, found for this sample to be in the range of 100–120 °C, can be explained by the stability of the immobilized [Ir(COD)Cl]_2_ complex linked to the surface of TiO_2_ by the N linker. At the same time, the increase in propene conversion in the same temperature range is the result of partial complex decomposition and iridium metal sintering. The total increase of the conversion at 120 °C up to 3% is rather small, an can be considered the result of the sintering of Ir and a drop in the Ir content available for the reaction. TEM data (see Figure 7) show the presence of both individual nanoparticles of Ir (a rounded bright object with size of ~1.2 nm in the middle-right of the image) and bright spots of atomic size. More HR TEM images are presented in Figure S4. It should be mentioned that no clusters similar to those in the Ir-P-TiO_2_ sample (Figure 4) are seen, which is in agreement with the differences in the catalytic behavior of these samples (Figure 1).

## 3. Materials and Methods

Immobilized metal complex preparation: For catalyst sample preparation, titanium dioxide, TiO_2_ (Hombifine N, S_BET_ = 350 m^2^/g) pre-calcined at 500 °C for 2 h (S_BET_ = 107 m^2^/g), 2-(4-pyridylethyl)triethoxysilane (95%, ABCR, cas: 98299-74-2, Figure 8a, labeled as C_2_H_4_Py in Table 1), 2-(diphenylphosphino)ethyltriethoxysilane (97%, ABCR, cas:18586-39-5, Figure 8b, labeled as C_2_H_4_P(Ph)_2_ in Table 1), (3-N,N-dimethylaminopropyl)triethoxysilane (97%, ABCR, cas: 43108-00-5, Figure 8c, labeled as C_3_H_6_N(CH_3_)_2_ in Table 1), bis[chloro(1,5-cyclooctadiene iridium(I)] [Ir_2_(COD)_2_(μ^2^-Cl)_2_] (99%, STREM Chemicals Inc., Newburyport, MA, USA), benzene (99.8%, Sigma Aldrich, Burlington, MA, USA), and ethanol (Pharm M, 95%) were used.

Modification of titanium oxide: Titanium dioxide, TiO_2_ (5 g), was dried at 120 °C for 12 h and evacuated. After that, 20 mL of benzene was added to the dried TiO_2_ with stirring. Then, 1 mL of silane was added to the resulting suspension (in the case of (diphenylphosphino)ethyltriethoxysilane, 0.5 mL was used). The reaction mixture was stirred for 14 h. After that, the powder was filtered off, washed with benzene and ethanol, and dried in a vacuum for 6 h. 

Anchoring of [Ir_2_(COD)_2_(μ^2^-Cl)_2_]: Briefly, 0.5 g of modified TiO_2_ and 65 mg of [Ir_2_(COD)_2_(μ^2^-Cl)_2_] were dried in a vacuum for 30 min, then 5 mL of benzene was added and the mixture was stirred for 3 h. Then, the sediment was filtered off, washed with benzene and ethanol and dried in a vacuum for 4 h. The description of the samples of catalysts is shown in Table 1. Details of the coordination of the complex compound [Ir_2_(COD)_2_(μ^2^-Cl)_2_] with the support are described elsewhere [2]. For the immobilized samples, the coordination of the [Ir_2_(COD)_2_(μ^2^-Cl)_2_] complex with the functional groups of the support took place and the stoichiometry of Ir/Cl = 1 remained unchanged.

For the catalytic reaction tests, samples of the catalysts (20 mg) were placed at the bottom of a standard NMR tube (10 mm), which was transferred to the NMR spectrometer. Propene hydrogenation was carried out under the strong magnetic field (7.1 T) (PASADENA experiment [11]) of a NMR spectrometer (Bruker AV 300 (300 MHz) in the 40–120 °C temperature range with a 20 °C step. 

The reaction mixture of propene (99.5%, “Pure gases”) and p-H_2_ (99.995%, “Alphagas”) in a 1:4 ratio was used for the hydrogenation reaction; in NMR experiments, two consecutively connected identical NMR tubes were used. The reaction mixture was continuously supplied through a glass capillary into the tube with the catalyst placed in the NMR spectrometer at a flow rate of 3.4 mL/s, and the ^1^H NMR spectra of the reaction mixture were recorded using a π/4-pulse. The reaction mixture was then directed to the second tube via a glass capillary. After the abrupt termination of the gas flow, the tubes switched places and ^1^H-NMR spectra of the products of the reaction in the second tube under equilibrium conditions were recorded using a π/2-pulse. 

Propene conversion (K) in all experiments was determined by comparing the integral intensity of the propene CH_3_ group NMR signal before and after the reaction as follows:

K = (1 − I_(after)_/I_(before)_) × 100%,

where I_(after)_ and I_(before)_ are the integral intensities of the CH_3_ group NMR signal of propene in thermal equilibrium after and before the reaction, respectively. 

The enhancement factor (EF) of the ^1^H NMR signal was calculated for the propane CH_3_ group in accordance with the following equation:

EF = ((I_(+1/2)_^(PHIP)^ + (−1) × (I_(−1/2)_^(PHIP)^)) × 6/I_(eq)_,

where I_(+1/2)_^(PHIP)^ and I_(−1/2)_^(PHIP)^ are the integral intensities of the absorption and emission components of the hyperpolarized NMR signal of the CH_3_ group of propane, and I_(eq)_ is the integral intensity of the CH_3_ group signal of propane under thermal equilibrium.

**Figure 9:**
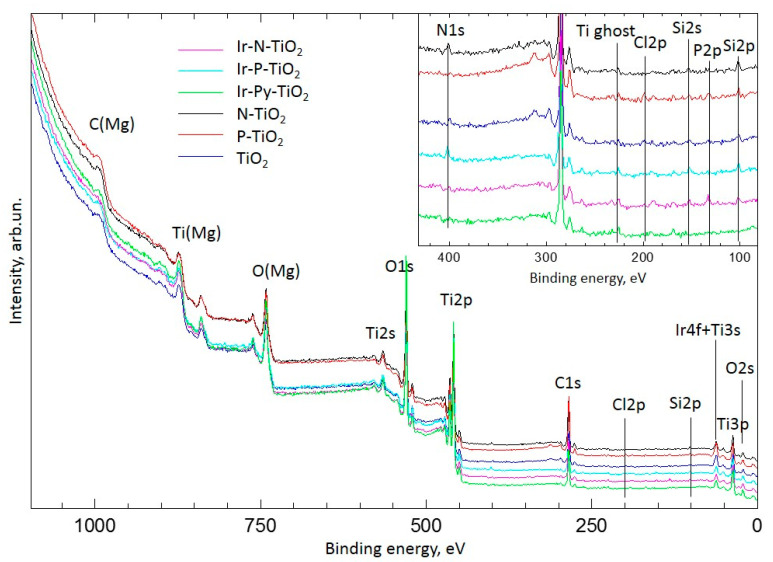
XPS survey spectra of studied samples.

The XPS study was carried out on a PHOIBOS-150/MCD-9 photoelectron spectrometer (SPECS Surface Nano Analysis GmbH), with non-monochromatized Mg*K*_α_ radiation and a power of 200 W. Before measurements, the energy scale of the spectrometer was calibrated to the positions of Au4f_7/2_ (84.0 eV) and Cu2p_3/2_ (932.7 eV) lines. The residual gas pressure in the analyzer chamber did not exceed 3 × 10^−7^ Pa during spectrum acquisition. The samples were fixed on the standard holder by pressing them into 3M^ΤΜ^ conductive Cu adhesive tape without further grinding. The Binding energy values and the areas of peaks for each XPS region were determined after the subtraction of the Shirley background and the analysis of line shapes. Curves were fitted with Gaussian–Lorentzian functions. For quantitative analysis, the atomic ratios calculated as the ratios of intensities of the main lines of elements to the elemental sensitivity factors with regard to the transmission function of the analyzer [23] were used. The spectra were processed using XPSPeak 4.1 software. High-resolution spectra are shown in Figures S5–S10. The errors are ~2% for the atomic ratios and 0.1 eV for the binding energy positions of the peaks. For all studied samples, the binding energy of Ti2p_3/2_ was 458.8 eV [23]. According to XPS, original titania contains traces of N, P and Si (Figure 9). These were taken into account in the quantitative analysis of the prepared samples of the immobilized Ir catalysts. The regions of Ti3s and Ir4f lines overlap, and these were taken into account for the decomposition of the spectra of catalysts based on the analysis of the Ti2p and Ti3s regions recorded for clean TiO_2_ and modified supports. 

The study conducted via transmission electron microscopy was performed using a high-resolution transmission electron microscope ThemisZ (Thermo Fisher Scientific, Waltham, MA, USA) with the lattice resolution of 0.07 nm and an accelerating voltage of 200 kV. The samples for this study were fixed on the standard copper meshes placed into the holder and put in the chamber of the electron microscope. The set-up was equipped with the SuperX energy-dispersive X-ray spectrometer (Thermo Fisher Scientific, Waltham, MA, USA) with a semiconductor Si detector with a 128 eV energy resolution. Computer vision and deep learning for microscopy image analysis were used [33].

## 4. Conclusions

The set of single-site [Ir(COD)Cl]_2_-Linker-TiO_2_ catalysts was synthesized, and their catalytic behavior in propene hydrogenation with parahydrogen in a 40–120 °C temperature range was investigated via NMR. The as-prepared samples and the samples after the reaction at 120 °C were studied via XPS. Based on the comparative analysis of reaction tests and XPS data, three patterns of catalytic behavior were observed and rationalized. For the Ir-Py-TiO_2_ sample, a sharp drop in NMR signal enhancement at 120 °C was associated with the partial removal of the 2-(4-pyridylethyl)triethoxysilane linker from the TiO_2_ surface and [Ir(COD)Cl]_2_ complex decomposition. The latter led to a rise in conversion due to the formation of highly dispersed metallic Ir under reaction conditions. For the Ir-P-TiO_2_ sample, the removal of the 2-(diphenylphosphino)ethyltriethoxysilane linker was also found. The apparent invariance in activity and the enhancement of NMR signal independently of the reaction temperature are attributed to the simultaneous action of two opposing trends: a rise due to the increasing temperature and a drop due to the partial removal of the [Ir(COD)Cl]_2_ complex from the catalyst surface together with the linker. For the Ir-N-TiO_2_ sample, the activation of the sample with the temperature increase can be explained by the stability of the (3-N,N-dimethylaminopropyl)triethoxysilane linker under reaction conditions. Hence, the [Ir(COD)Cl]_2_ complex anchored by this linker to TiO_2_ is responsible for the rise in enhancement, while partial complex decomposition explains the rise in conversion. 

Thus, the reconciliation of reaction data with XPS data is effective for the understanding of the catalytic behavior of the single-site catalysts.

## Figures and Tables

**Figure 8:**
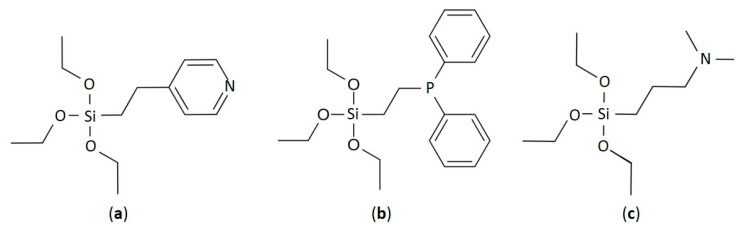
Chemical structures of the linkers: (**a**) 2-(4-pyridylethyl)triethoxysilane; (**b**) 2-(diphenylphosphino)ethyltriethoxysilane; (**c**) (3-N,N-dimethylaminopropyl)triethoxysilane.

**Table 1:** The list of the catalysts immobilized on TiO_2_ that were synthesized and studied in this work.

Sample	Linker	Complex Compound
Ir-Py-TiO_2_	C_2_H_4_Py	[Ir(COD)Cl]_2_
Ir-P-TiO_2_	C_2_H_4_P(Ph)_2_	[Ir(COD)Cl]_2_
Ir-N-TiO_2_	C_3_H_6_N(CH_3_)_2_	[Ir(COD)Cl]_2_

## Data Availability

Not applicable.

## Supplementary Materials

The following supporting information can be downloaded at: https://www.mdpi.com/article/10.3390/ijms242115643/s1.
