# Supplementary material for: XPS and HR TEM Elucidation of the Diversity of Titania-Supported Single-Site Ir Catalyst Performance in Spin-Selective Propene Hydrogenation
Source: Int J Mol Sci. 2023 Oct 27;24(21):15643. doi: 10.3390/ijms242115643 (PMC10650017; doi:10.3390/ijms242115643)
Supplement: Supplementary file 1 [file ijms-24-15643-s001.zip › ijms-2653704-supplementary.pdf]

Supplementary Materials

XPS and HR TEM Elucidation of the Diversity of Titania-Supported Single-Site Ir Catalyst Performance in Spin-Selective Propene Hydrogenation

Anna V. Nartova [1]*, Ren I. Kvon [1], Larisa M. Kovtunova [1], Ivan V. Skovpin [2], Igor V. Koptyug [2] and Valerii I. Bukhtiyarov [1]

[1] Department of Physical-Chemical Methods of Investigation, Boreskov Institute of Catalysis SB RAS, Lavrentiev Ave. 5, 630090 Novosibirsk, Russia; kvon@catalysis.ru (R.I.K.); kovtunova@catalysis.ru (L.M.K.); vib@catalysis.ru (V.I.B.)

[2] Laboratory of Magnetic Resonance Microimaging, International Tomography Center SB RAS, Institutskaya St. 3A, 630090 Novosibirsk, Russia; iskovpin@tomo.nsc.ru (I.V.S.); koptyug@tomo.nsc.ru (I.V.K.)

* Correspondence: nartova@catalysis.ru (A.V.N.); Tel.: +7-3833269633

NMR experimental data are shown in Figures S1, S2, S3.

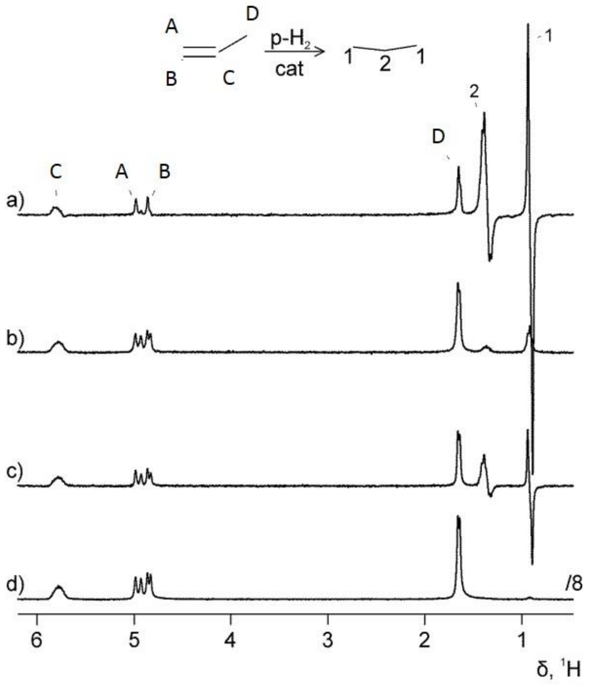

Figure S1. ^{1}H NMR PHIP spectra recorded with propane/p-H_2 mixture flowing (3.4 ml/s) through Ir – Py – TiO_2 at (a) 120°C, (c) 100°C. Thermal equilibrium ^{1}H NMR spectra (b, d) were recorded on the corresponding reaction mixture (a and c, respectively) after relaxation of the hyperpolarized propene. Spectra (a, c, d) were acquired with one signal accumulations, (b) with eight signal accumulations; they are scaled accordingly and are presented on the same vertical scale.

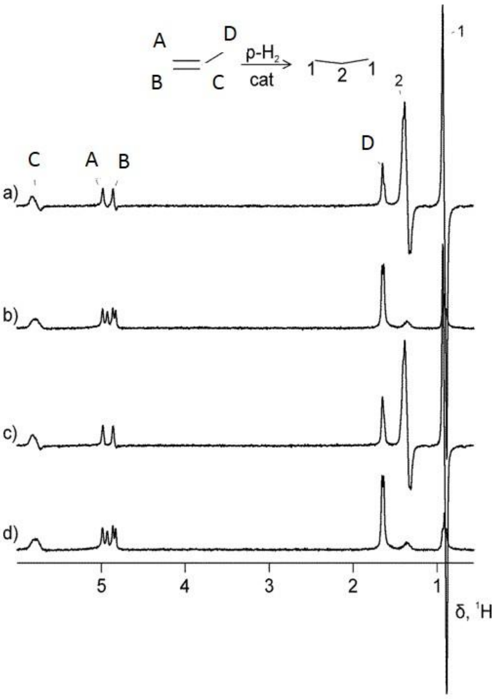

Figure S2. ^{1}H NMR PHIP spectra recorded with propane/p-H_2 mixture flowing (3.4 ml/s) through Ir - P - TiO_2 at (a) 120°C, (c) 100°C. Thermal equilibrium ^{1}H NMR spectra (b, d) were recorded on the corresponding reaction mixture (a and c, respectively) after relaxation of the hyperpolarized propene. Spectra (a, b, c and d) were acquired with one signal accumulations.

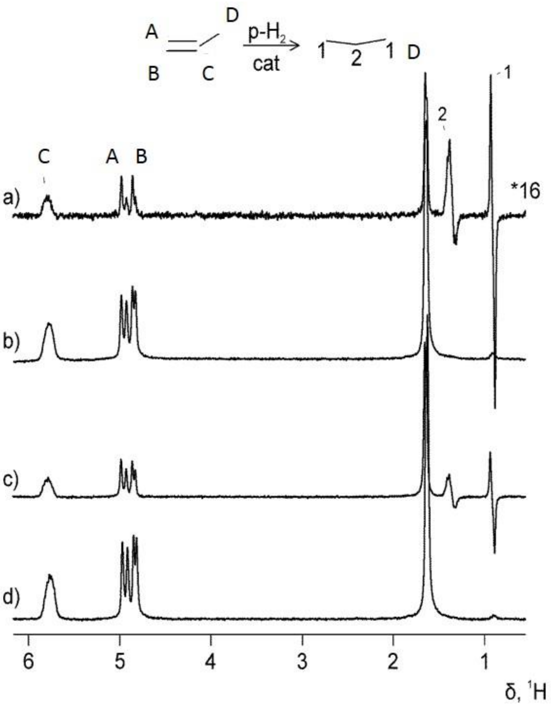

Figure S3. ^{1}H NMR PHIP spectra recorded with propane/p-H_2 mixture flowing (3.4 ml/s) through Ir - N - TiO_2 at (a) 120°C, (c) 100°C. Thermal equilibrium ^{1}H NMR spectra (b, d) were recorded on the corresponding reaction mixture (a and c, respectively) after relaxation of the hyperpolarized propene. Spectra (b, c, d) were acquired with sixteen signal accumulations, (a) with one signal accumulations; they are scaled accordingly and are presented on the same vertical scale.

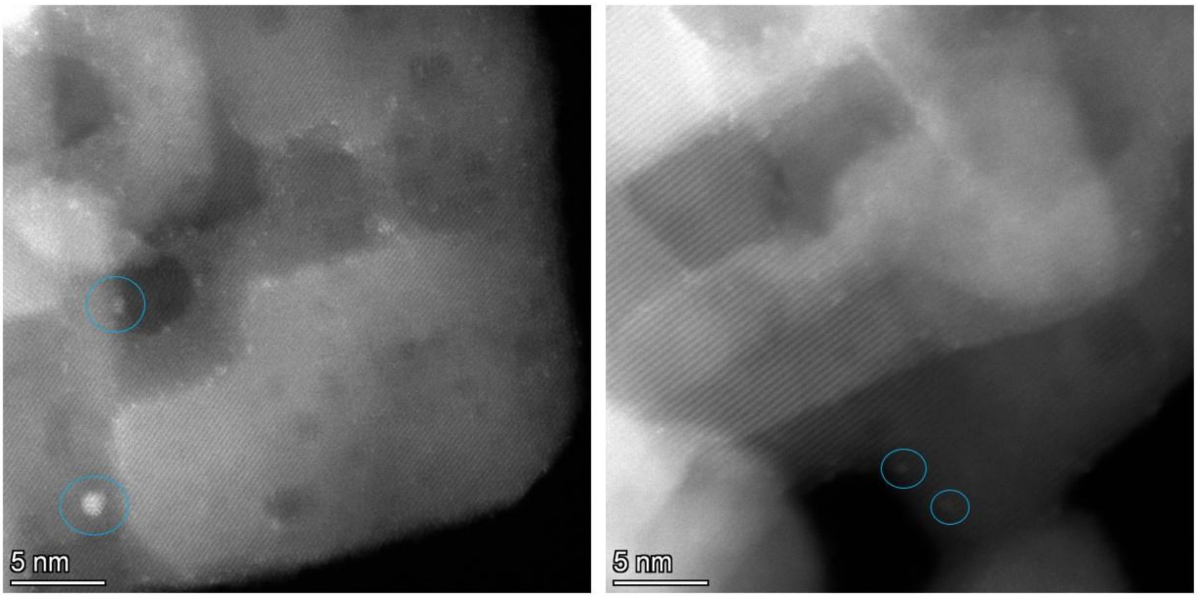

Figure S4. TEM data for the Ir-N-TiO_2 sample after reaction at 120°C (Ir nanoparticles are marked by circles).

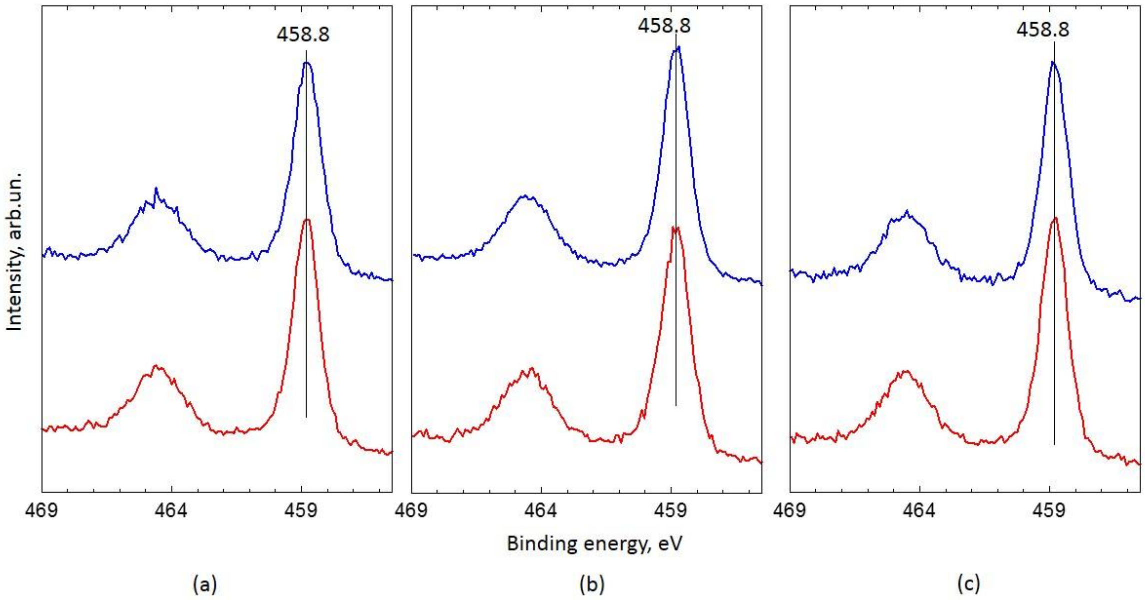

Figure S5. XPS of the Ti 2p line before (top blue) and after (bottom red) propene hydrogenation reaction at 120°C: (a) for the Ir-Py-TiO_2 sample; (b) for the Ir-P-TiO_2 sample; (c) for the Ir-N-TiO_2 sample.

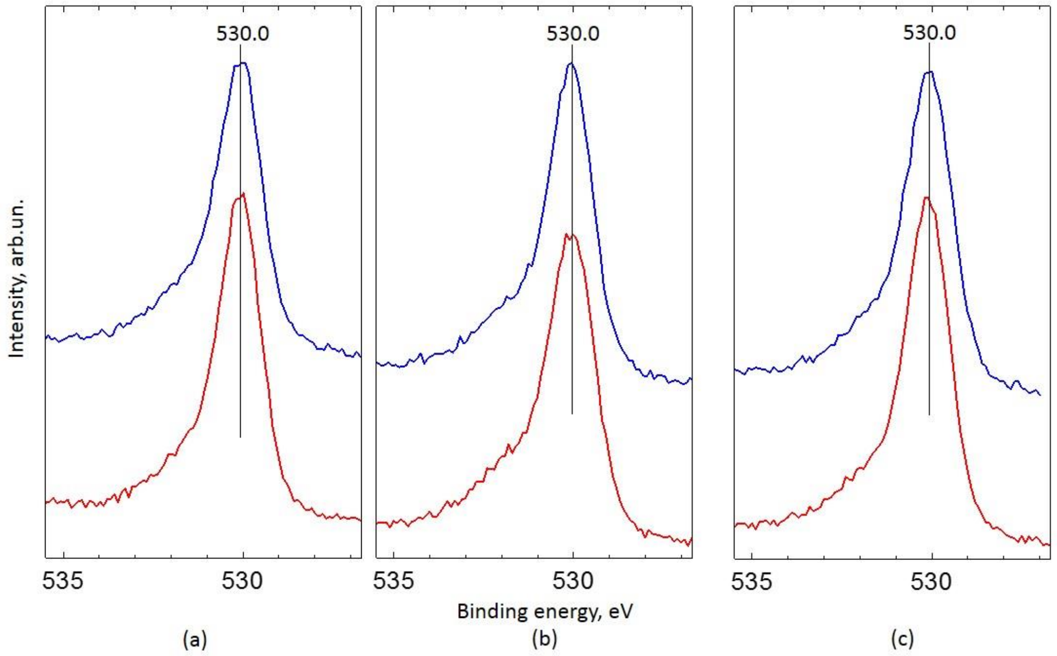

Figure S6. XPS spectra of the O 1s line before (top blue) and after (bottom red) propene hydrogenation reaction at 120°C: (a) for the Ir-Py-TiO_2 sample; (b) for the Ir-P-TiO_2 sample; (c) for the Ir-N-TiO_2 sample.

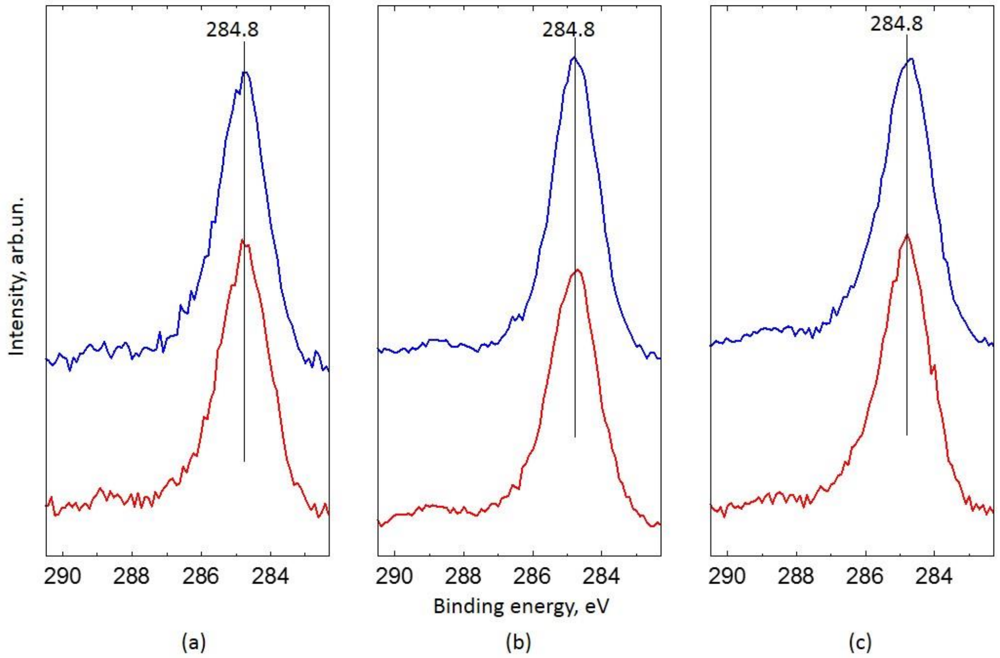

Figure S7. XPS spectra of the C 1s line before (top blue) and after (bottom red) propene hydrogenation reaction at 120°C: (a) for the Ir-Py-TiO_2 sample; (b) for the Ir-P-TiO_2 sample; (c) for the Ir-N-TiO_2 sample.

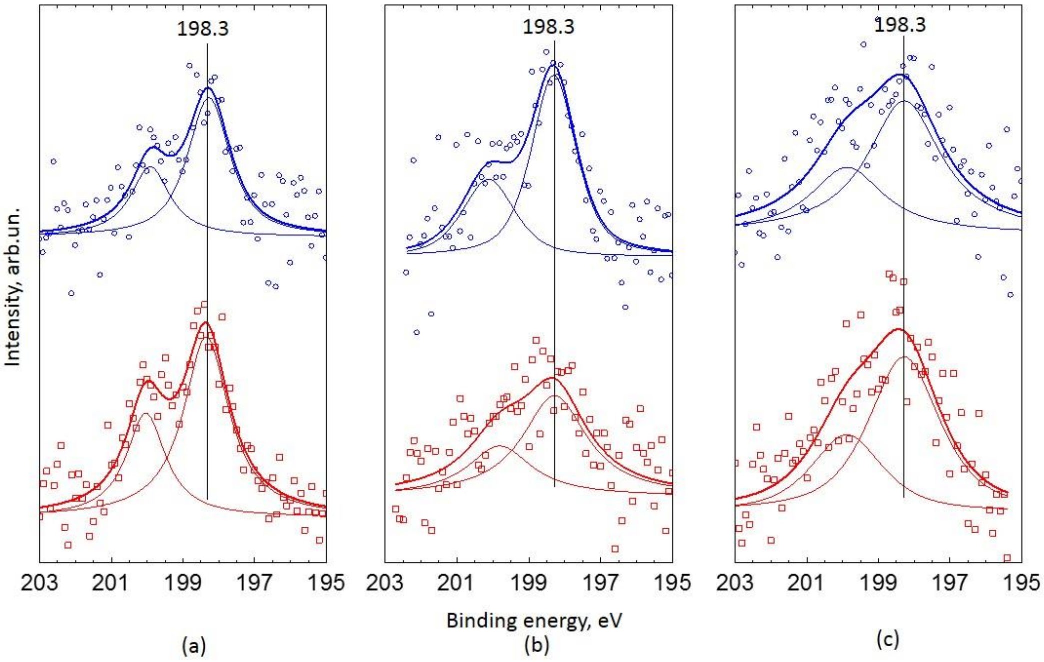

Figure S8. XPS spectra of the Cl 2p line before (top blue, experimental spectra (points) and spectrum deconvolutions (solid lines)) and after (bottom red, experimental spectra (points) and spectrum deconvolutions (solid lines)) propene hydrogenation reaction at 120°C: (a) for the Ir-Py-TiO_2 sample; (b) for the Ir-P-TiO_2 sample; (c) for the Ir-N-TiO_2 sample.

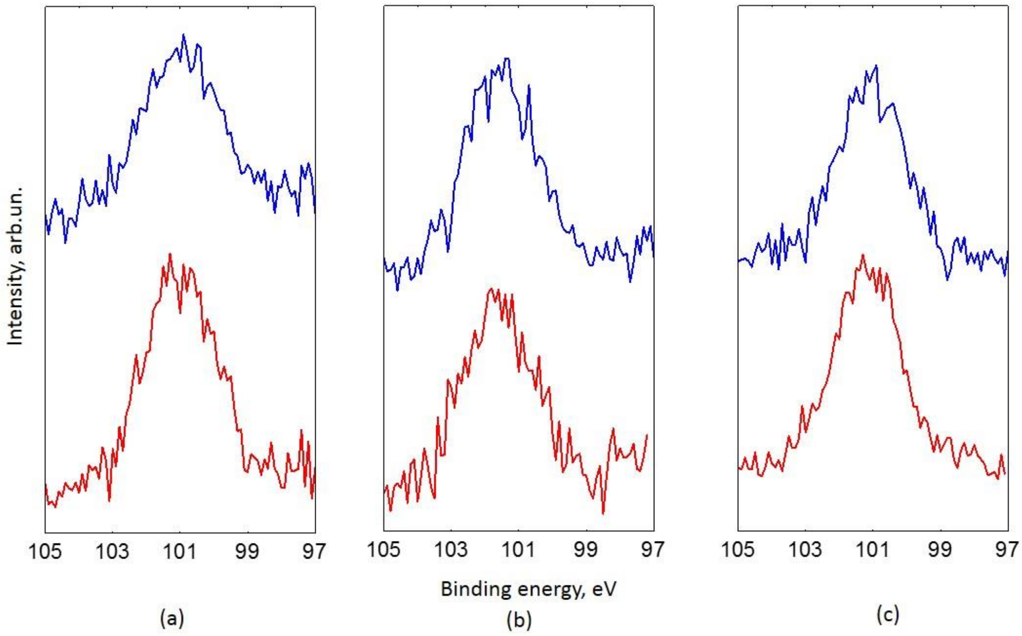

Figure S9. XPS spectra of the Si 2p line before (top blue) and after (bottom red) propene hydrogenation reaction at 120°C: (a) for the Ir-Py-TiO_2 sample; (b) for the Ir-P-TiO_2 sample; (c) for the Ir-N-TiO_2 sample.

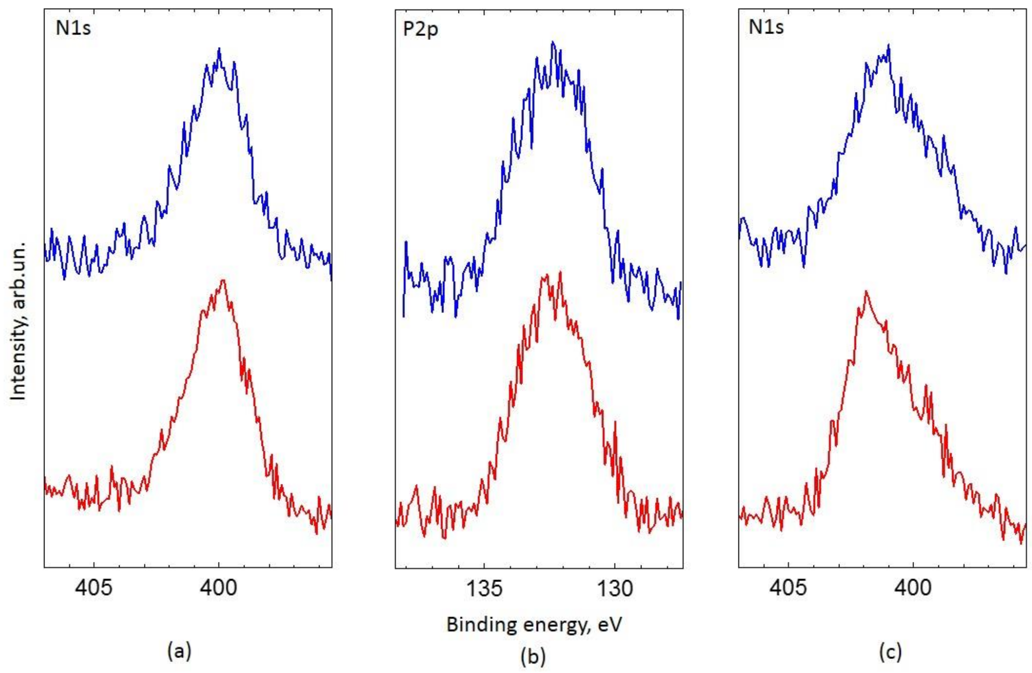

Figure S10. XPS spectra of the lines of heteroatoms of the Linkers before (top blue) and after (bottom red) propene hydrogenation reaction at 120°C: (a) N1s for the Ir-Py-TiO_2 sample; (b) P2p for the Ir-P-TiO_2 sample; (c) N1s for the Ir-N-TiO_2 sample.
